# Screening for Zika virus RNA in sera of suspected cases: a retrospective cross-sectional study

**DOI:** 10.1186/s12985-018-1070-z

**Published:** 2018-10-11

**Authors:** Lívia Sacchetto, Danielle Alves Gomes Zauli, Galileu Barbosa Costa, Sarah Anne J Guagliardo, Luige Biciati Alvim, Feliciana Lage de Oliveira Marinho, Jônatas Santos Abrahão, Giliane de Souza Trindade, Erna Geessien Kroon, Elvis Cristian Cueva Mateo, Betânia Paiva Drumond

**Affiliations:** 1 Laboratório de Vírus, Departamento de Microbiologia, Instituto de Ciências Biológicas, Universidade Federal de Minas Gerais, Bloco F4, Sala 258, Avenida Antônio Carlos, 6627, Belo Horizonte, Minas Gerais 31270-901 Brazil; 2 Instituto Hermes Pardini, Vespasiano, Minas Gerais Brazil; 3 Poxvirus and Rabies Branch, US Centers for Disease Control and Prevention, Atlanta, GA USA; 4 Epidemic Intelligence Service, US Centers for Disease Control and Prevention, Atlanta, GA USA; 5 Programa de Pós-graduação em Ciências Biológicas, Universidade Federal de Juiz de Fora, Juiz de Fora, Minas Gerais Brazil

**Keywords:** Zika virus, Cross-sectional study, Epidemiology, Brazil, Dengue virus, Chikungunya virus

## Abstract

**Background:**

Zika virus (ZIKV) became a global human health concern owing to its rapid spread worldwide and its association with congenital and neurological disorders. The current epidemiological profile of arboviruses in Brazil is characterized by widespread co-circulation of Dengue virus, Chikungunya virus, and ZIKV throughout the country. These viruses cause acute diseases frequently with overlapping symptoms, which could result in an inaccurate diagnosis based solely on clinical and epidemiological grounds. Here we conducted a screening for ZIKV RNA in serum samples from patients across Brazil with suspected ZIKV infection.

**Methods:**

Using RT-qPCR, we investigated ZIKV RNA in 3001 serum samples. Samples were passively acquired through a private laboratory network, between December 2015 and August 2016, from 27 Brazilian Federative Units. We performed descriptive statistics on demographic variables including sex, age, and geographic location.

**Results:**

ZIKV was detected in 11.4% (95%CI = 10.3–12.6%) of the sera. ZIKV RNA was detected in sera collected throughout the country, but during the analyzed period, RNA was more frequently detected in samples from the Southeast, Midwest, and North regions (3.9 to 5.8 times higher) when compared to the Northeast and South regions.

**Conclusions:**

These data reinforce the importance of laboratory diagnosis, surveillance systems, and further epidemiological studies to understand the dynamics of outbreaks and diseases associated with ZIKV and other arboviruses.

**Electronic supplementary material:**

The online version of this article (10.1186/s12985-018-1070-z) contains supplementary material, which is available to authorized users.

## Background

The genus *Flavivirus* of the family *Flaviviridae* contains several well-known mosquito-borne viruses of medical importance, including Zika virus (ZIKV), Dengue virus (DENV), West Nile virus, Japanese encephalitis virus, and Yellow fever virus [1, 2]. ZIKV was first isolated in 1947 in the Zika Forest of Uganda, and for most of the virus’s history, evidence of infections came mostly from sporadic serologic studies in Africa and Asia [3]. In recent years, ZIKV has spread to 84 countries and territories in the Caribbean region, Latin America, and North America [4].

The most common symptoms of ZIKV infection are fever (usually lower than 38.5 °C), pruritic and maculopapular rash, joint pain, periarticular edema, and conjunctivitis (non-purulent/hyperaemic), muscle pain, and headache [5, 6]. Most ZIKV infections are mild or asymptomatic [2, 3, 6], but severe complications have been observed, as fetal microcephaly and neurologic symptoms such as Guillain-Barré syndrome [2, 3, 7]. ZIKV infection suspected cases had been defined by Brazilian Health authorities as patients having a pruritic and maculopapular rash along with two or more of the following symptoms: fever, conjunctival hyperemia (non-purulent), polyarthralgia and periarticular edema [8]. According to the Brazilian Ministry of Health, confirmed cases are defined as a suspected case with a positive result in one test for ZIKV infection diagnoses, such as virus isolation, detection of viral RNA by reverse transcription followed by polymerase chain reaction, or by a serologic test to detect IgM antibodies. However, after the confirmation of viral autochthonous circulation in an area, case confirmation may take place only by clinical/epidemiological criteria, except for pregnant women, patients with neurologic disorders, and deaths [8]. It is important to keep in mind that given cross-reactions between flaviviruses, the serology results are difficult to be interpreted in patients from endemic areas or in patients with history of vaccination against flaviviruses. The best alternative would be the molecular detection of viral RNA, during the acute phase. Whole blood and urine have been proposed as best samples since the virus can be detected only briefly in plasma or serum during acute illness [3].

In Brazil, ZIKV was first reported in March of 2015 in Rio Grande do Norte and Bahia states located in the Northeast region [9, 10] (Additional file 1: Figure S1) with subsequent spread throughout the country. At the end of 2015, autochthonous circulation of ZIKV was confirmed in 19 states of Brazil, with wide dissemination in the Northeast regions (eight out of the nine states) [11]. From data from the Brazilian Ministry of Health, up to the epidemiological week (EW) 52 of 2016, ZIKV circulation was reported in all 27 Brazilian Federated Units (see Additional file 2: Table S1 and Additional file 3: Table S2 for definition of EW). In 2016, 215,319 suspected cases were reported with 130,701 confirmed cases, although there is no information whether the cases were confirmed by laboratory tests or solely by clinical-epidemiological criteria [12]. The highest incidence rate was observed in the Midwest region with 222.0 cases/100,000 inhabitants, followed by the Northeast and Southeast regions with 134.4 and 106.2 cases/100,000 inhabitants, respectively (Official data were retrieved from bulletin published by the Brazilian Ministry of Health and are presented in Additional file 2: Table S1) [12]. The incidence rates of ZIKV infection in 2017 decreased from 5.5 (Midwest region) to 25 times lower (Southeast region) when compared to the incidences rates observed in 2016, indicating a decrease in virus circulation all over the country from 2016 to 2017 (Official data were retrieved from bulletins published by Brazilian Ministry of Health and are presented in Additional file 3: Table S2).

The epidemiological scenario in Brazil during ZIKV outbreaks was characterized by well-documented co-circulation of DENV and Chikungunya virus (CHIKV), among other arboviruses. In 2016, up to EW 52, 271,824 suspected chikungunya cases and 1,500,535 suspected cases of dengue were reported (Official data were retrieved from bulletins published by the Brazilian Ministry of Health and are presented in Additional file 2: Table S1) [12]. Infection with DENV and CHIKV can lead to asymptomatic, mild, or severe disease, and symptoms may be similar to ZIKV infection including headache, joint pain, and rash [13, 14]. Thus, the diagnosis of dengue, Zika, and chikungunya infections based on clinical criteria may frequently be confused, reinforcing the need for laboratory diagnosis for case confirmation [2, 3, 15]. Given the emergent nature and the severity of disease associated with ZIKV infection during simultaneous dengue and chikungunya outbreaks in Brazil, the aim of this study was to contribute to the molecular screening of ZIKV in suspected acute cases from across Brazil.

## Materials and methods

### Patients and clinical samples

A retrospective cross-sectional screening of ZIKV RNA in serum samples passively acquired through a private laboratory network in Brazil was performed. Samples were received in a private diagnostic medicine laboratory (Hermes Pardini Institute – HP Institute). In that way, a convenience sampling, a type of non-probability sampling, which does not include random selection of participants, was used in this study. HP Institute has a network of laboratories all over Brazil that conducts testing for a variety of infectious and non-infectious diseases. The Institute has a national logistics operation that guarantees cold-chain maintenance and an average transit time of 19 h from the shipment to the arrival of the samples at the operational headquarters, in Belo Horizonte, for diagnosis. The samples were collected, processed, and frozen according to standard procedures, before being sent to the HP Institute. Inadequately preserved samples were discarded and excluded from the study. The serum samples included in the study were received from ZIKV infection suspected patients based on the symptoms [8], living in all 27 Federated Units of Brazil and collected up to five days of the onset of the symptoms. Information regarding age, EW/year of sampling and also geographic origin of each participant were taken from patient’s file (Additional file 4: Table S3). Further information on the patients’ clinical symptoms and demographic information were not available. The samples were collected from patients living in all 27 Federated Units of Brazil but given the convenience sampling, they were mostly received from the Southeast, followed by the Northeast and Midwest regions of Brazil. The study period, from December 2015 to August 2016, was defined based on the availability of samples received for analysis. As such, a total of 3001 sera fitted the criteria described above and were included in the study.

### RT-qPCR

Total viral RNA was extracted from 140-μL biological specimens using the QIAamp viral RNA mini kit (Qiagen, USA) according to the manufacturer’s instructions. Detection of ZIKV RNA was performed by reverse transcription followed by real-time polymerase chain reaction (RT-qPCR) with five μl of total RNA, primers and TaqMan fluorescent probes targeting the NS5 region of the ZIKV RNA, provided in the Bio Gene *Zika Virus* PCR Kit (Bioclin-Quibasa, Brazil), in a final volume of 20 μL. Each RT-qPCR run included non-template, negative, and internal controls in addition to a quantitative standard curve according to the manufacturer’s instructions. The internal control, provided in the kit, was added to each tube (containing the samples, the positive or the negative controls), previously to the RNA extraction. According to the manufacturers, the kit showed clinical sensitivity of 99.9% and a clinical specificity of 99.9%. The limit of detection is 10 copies of genome copy equivalents per reaction and the kit can detect up to 1,000,000 copies of genome copy equivalents per reaction [16]. This kit is approved by the Agência Nacional de Vigilância Sanitária (ANVISA), a Brazilian regulatory agency, under the registration number 10269360300.

### Statistical analysis

We performed descriptive statistical analysis, using the following information: sex, age and geographic origin of participants. Information regarding sex and age were not available from three and six participants, respectively, and these participants were not included in the analysis of those parameters. Participants were categorized as pediatrics (< 1–11 months, and 1–10 years), young adults (11–20 and 21–30 years), and adults (31–40, 41–50, and older than 50 years). Regarding the geographic origin, participants were categorized as from North, Northeast, South, Southeast, and Midwest regions (states in each region are provided in Table 1). Associations between these characteristics and ZIKV detection were examined using Chi-square and Fisher’s exact tests, with a significance cut-off of α = 0.05. Analyses were performed using Epi-Info 7.2 (EpiInfo™, GA, USA).

**Table 1:** Demographic variables of participants tested for ZIKV

Demographics	N (%)^**^	RT-qPCR positive (%)	RT-qPCR negative (%)	OR 95% CI	*p* value
Sex					
Female	2111 (70.3)	268 (12.7)	1843 (87.3)	1.59 (1.22–2.10)	0.0005^***^
Male^*^	887 (29.5)	74 (8.3)	813 (91.6)		
Age group					
< 1–11 m^*‡^	99 (3.3)	7 (7.1)	92 (92.9)		0.90
1–10 y	237 (7.9)	16 (6.8)	221 (93.2)		0.83
11–20 y	179 (5.9)	14 (7.8)	165 (92.8)		0.07
21–30 y	536 (17.8)	71 (13.2)	465 (86.8)		0.10
31–40 y	1007 (33.5)	126 (12.5)	881 (87.5)		0.21
41–50 y	287 (9.5)	33 (11.5)	254 (88.5)		0.187
> 50 y	645 (21.5)	74 (11.5)	571 (88.5)		
Brazilian regions					
South^*^	201 (6.7)	7 (3.4)	194 (96.5)		< 0.0001†
North	216 (7.2)	35 (16.2)	181 (83.8)	5.35 (2.32–12.36)	
Southeast	1240 (41.3)	175 (14.1)	1065 (85.8)	4.55 (2.10–9.84)	
Midwest	577 (19.2)	94 (16.2)	483 (83.7)	5.39 (2.45–11.83)	
Northeast	767 (25.5)	31 (4.0)	736 (95.9)	1.16 (0.50–2.69)	

^*^reference categories to which the other categories were compared to. m: months. y: years. ^**^Total may not add up 100% due to missing data. RT-qPCR: reverse transcription followed by real-time polymerase chain reaction. OR: Odds ratio. 95% CI: 95% confidence interval. ^***^Analysis performed using Fisher’s exact test. ^‡^Individuals included in this category are those with age ranging from < 1 month to 11 months. †Analysis performed using Chi-square for trend. Brazilian states in each region are as follows: South: Rio Grande do Sul, Paraná, and Santa Catarina; North: Acre, Amapá, Amazonas, Pará, Rondônia, Roraima, and Tocantins; Southeast: Minas Gerais, São Paulo, Rio de Janeiro and Espírito Santo; Midwest: Mato Grosso, Mato Grosso do Sul, Goiás and Distrito Federal; Northeast: Alagoas, Bahia, Ceará, Maranhão, Paraíba, Pernambuco, Piauí, Rio Grande do Norte and Sergipe

### Ethical statement

The study was approved by the Human Subjects Research Ethics Committee of the Federal University of Juiz de Fora (CAAE 3135216.9.0000.5147). The Committee approved the analysis of biological samples and laboratory files without the informed consent from each participant since the samples were collected and received for viral investigation from several laboratories in different regions of the country, and all data were analyzed anonymously with total confidentiality of each participant.

## Results

**Fig. 1:**
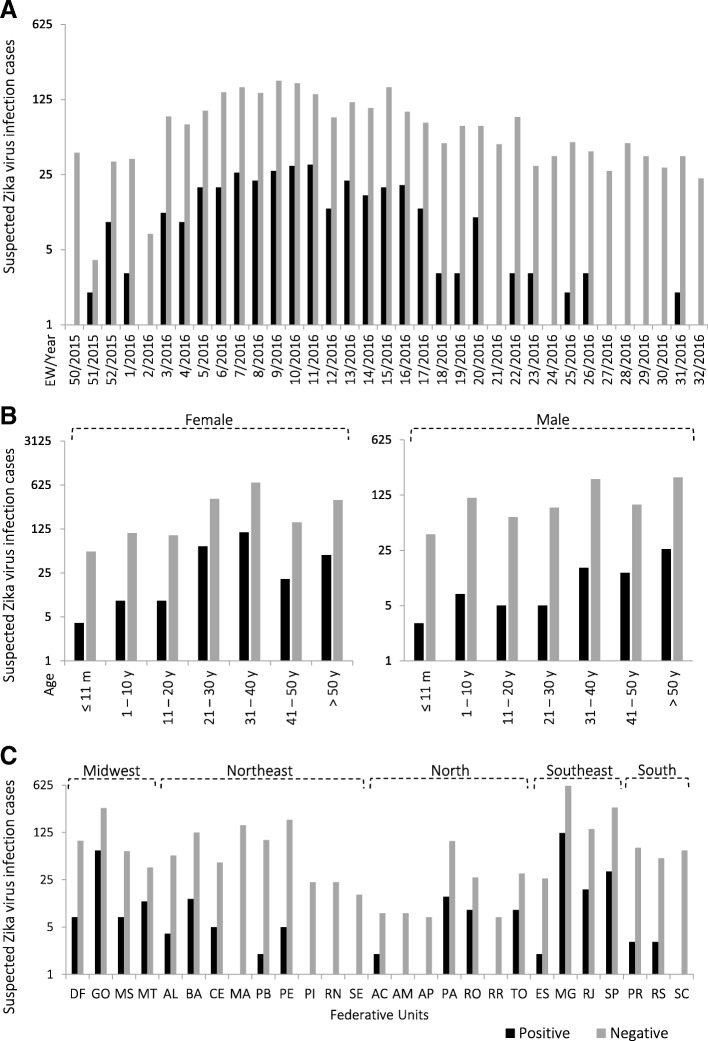
Molecular investigation of Zika virus in Brazil. A total of 3001 sera were tested by RT-qPCR between December 2015 and August 2016. **a** Positive and negative samples per epidemiological week (EW) and year. **b** Positive and negative samples according to sex and age (m: months. y: years). **c** Distribution of positive and negative samples according to Federative Units and regions. Positive and negative samples are shown in black and gray, respectively. Brazilian Federative Units are indicated as follows: DF: Distrito Federal; GO: Goiás; MS: Mato Grosso do Sul; MT: Mato Grosso; AC: Acre; AM: Amazonas; AP: Amapá; PA: Pará; RO: Rondônia; RR: Roraima; TO: Tocantins; AL: Alagoas; BA: Bahia; CE: Ceará; MA: Maranhão; PB: Paraíba; PE: Pernambuco; PI: Piauí; RN: Rio Grande do Norte; SE: Sergipe; PR: Paraná; RS: Rio Grande do Sul; SC: Santa Catarina; ES: Espírito Santo; MG: Minas Gerais; RJ: Rio de Janeiro; SP: São Paulo

In the present study, 3001 serum samples from ZIKV infection suspected patients were received over a nine-month period were analyzed by RT-qPCR to detect ZIKV infection. Most samples (78.5%; 2357/3001) were collected between EW 3/2016 and EW 20/2016 (Fig. 1a) and also the majority of the positive sera (90.9%; 311/342) was collected in this time period. The majority of the patients were women (70.3%; 2111/3001), approximately half were from individuals aged 21–40 years (51.4%; 1543/3001) (Table 1, Fig. 1b), and 41.3% (1240/3001) of the samples were from the Southeast region of Brazil, especially the states of Minas Gerais and São Paulo (Table 1, Fig. 1c).

Of the 3001 sera, 342 tested positive for ZIKV RNA (Cycle threshold (Ct) cut-off = 36), representing a positivity rate of 11.4% (95% confidence interval [95% CI] = 10.3–12.6%). All samples were positive for the internal control (Ct cutoff = 32). The detection of ZIKV RNA in the screening was 1.59 times higher for women than for men (odds ratio [OR] = 1.59; 95% CI = 1.22–2.10; *p* = 0.0005] (Table 1). No statistical difference regarding the detection of ZIKV RNA was observed between age groups (*p* ≥ 0.07) (Table 1).

ZIKV RNA was detected in sera from patients living in all regions of Brazil (Table 1) in 23 out of 27 Brazilian Federated Units (Fig. 1c), and the detection of ZIKV RNA in samples from the Southeast, Midwest, and North regions was 3.9 to 5.8 times higher than those from the Northeast and South regions (Additional file 5: Table S4). No differences were observed in ZIKV positivity between the Southeast, Midwest, and North regions (*p* ≥ 0.25) or between the South and Northeast regions (*p* = 0.83) (Additional file 5: Table S4).

## Discussion

Since the first registered case of ZIKV in Brazil in 2015 [9, 10] the virus spread rapidly throughout the country [11]. In this study, we performed a screening for ZIKV RNA in sera collected from ZIKV infection suspected acute cases seeking health care, from throughout the country through a private laboratory network. We observed an overall positivity rate of ZIKV RNA in 11.4% of the samples. The majority of the positive samples were collected between EW 3/2016 and EW 20/2016, coinciding with the period that the greatest numbers of ZIKV infection suspected cases were registered in 2016 [17], within the epidemic season of arboviruses in Brazil. Our positivity estimates of ZIKV infection (11.4%) were significantly lower than the prevalence estimate of the Brazilian Ministry of Health (51.7%) for the same period [18]. The official Brazilian Ministry of Health bulletins reporting ZIKV infection suspected and confirmed cases are also based on convenience sampling, represented by people presenting symptoms and seeking health care assistance. The ZIKV infection cases can be confirmed based on laboratory diagnosis, clinical or epidemiological criteria [8]. In that way, different symptoms presented by the patients and different criteria for case confirmation may explain the differences observed between our results (based on RT-qPCR) and those from the Brazilian Ministry of Health (based on RT-PCR, serology tests, or only on clinical-epidemiological criteria). Unfortunately, there is no information about the tests run by the Brazilian Ministry of Health, or regarding how many cases were laboratory confirmed by the Brazilian Ministry of Health laboratories, based on serology or molecular approaches [12, 18]. In addition, this difference could also be explained by sampling bias associated with data passively acquired through a laboratory network or even by clinical misdiagnosis of some patients with acute febrile disease.

ZIKV was detected in all regions of Brazil corroborating data from the Brazilian Ministry of Health [12]. However, in contrast to the official reports [12], we observed a lower occurrence of ZIKV in Northeast region compared to the Midwest, Southeast, and North regions. Lourenço and colleagues [19], using an ento-epidemiological transmission model, analyzed the Zika outbreak (from 2015 up to 2017) in one major urban center (Feira de Santana, Bahia state) in the Northeast region. The authors observed high attack rates during the first wave of the epidemics when 65% of Feira de Santana population were estimated to have been infected by ZIKV up to the end of 2015 [19]. The authors demonstrated that the high transmission potential of ZIKV in an urban center could lead to the exhaustion of susceptible hosts, explaining the decline in ZIKV infection cases observed in the following years (2016 and 2017), due to herd immunity. Epidemics of ZIKV infection in Brazil began in the Northeastern region, which has been described as one of the most affected areas by ZIKV [11, 12] and had its peak in 2015. Then, in 2016, ZIKV infection was largely disseminated in Brazil, but incidence rates presented a significant drop in 2017 [20] (Additional file 3: Table S2). These data altogether could in part, explain the lower occurrence of ZIKV infection that we observed in the Northeast region, in 2016, compared to the Southeast, North and, Midwest regions of Brazil.

It is also important to note that in Brazil (up to EW 32 of 2016), 87.8% and 21.7% of the suspected cases of CHIKV and DENV infections, respectively, were reported in the Northeast region [18]. The co-circulation of DENV and CHIKV could lead to clinical misdiagnosis resulting in an increased number of samples, especially from the Northeast region, being sent for laboratory diagnosis of Zika. In fact, we observed a higher ratio of ZIKV infection suspected cases over the ZIKV infection laboratory-confirmed cases for participants from the Northeast region when compared to participants from other regions. This could also stem from concerns about the burden of ZIKV and the greatest numbers of associated neurological disorders in the Northeast region of the country, during Zika outbreaks [21].

The preponderance of young adults, especially women, sampled in this study could stem from a greater likelihood for women to seek health care, even with mild symptoms, and especially due to the concerns regarding sexual transmission of ZIKV and the association of ZIKV infection during pregnancy with microcephaly and other neurological disorders [2, 3, 7]. As previously observed [22, 23], we detected a higher number of ZIKV RNA positive samples among women. It has been proposed that higher infection rates in women could be due to greater exposure to the vector in the household environment [23].

Although some studies highlight the role of urine and whole blood as a source of sample for ZIKV diagnostics since the virus has been detected in higher titers than in serum for prolonged times [3], we were only able to test sera using RT-qPCR. It was mandatory to collect samples within the five days of the onset of the symptoms when the virus can be detected in serum samples, but we cannot rule out the low viremia in some samples, by the time that patients have symptoms, or shortly after that. On the other hand, given the logistics used, the external effect of sample collection and processing over the positivity rate was minimized, given the high-quality standard of sample collection, transport, and processing. Further, specimens used for this analysis were passively acquired through an existing laboratory network (i.e., convenience sampling) and were received for routine diagnosis from a single period (December 2015 to August 2016). Convenience sampling limits the interpretation of our data in that way, our results may not be representative of broader patterns of ZIKV infection in the general population. Detailed data on patient socioeconomic status and knowledge about ZIKV infection was not available, but these associations could perhaps be explored in future analyses regarding the use of passively-acquired data from laboratory networks.

It is also important to note that the overlapping ZIKV infection in clinical presentation with other arboviruses may artificially conflate diagnoses made solely on clinical/epidemiological criteria. In 2016, widespread dengue and chikungunya outbreaks took place in Brazil with more than 1.6 million suspected cases [12]. Although we were not able to test the samples for other arboviruses, some of the suspected cases that were ZIKV-negative could also represent clinical misdiagnoses of other infections, including the ones caused by other arboviruses such as DENV and CHIKV. The clinical misdiagnosis of diseases caused by ZIKV, DENV and CHIKV have already been demonstrated in some parts of Brazil, like São Paulo, Bahia, and Rio de Janeiro states [22–26] and our results indicate that this phenomenon might have happened throughout the country.

## Conclusions

Given the epidemiological context, laboratory differential diagnosis, distinguishing ZIKV from other arboviruses is imperative, given the widespread co-circulation of these viruses in Brazil and the fact that syndromic surveillance is not enough to accurately define the etiology of many acute exanthematous illnesses. ZIKV constitutes a public health burden, and future studies are crucial to the understanding of outbreak dynamics and disease associated with ZIKV infection, as well to the establishment of control and prevention measures, including any future vaccination programs.

## Additional files

Additional file 1: **Figure S1.** Geopolitical map of Brazil. The Rio Grande do Norte and Bahia states, where Zika virus was first reported, is highlighted. Brazilian Federative Units are indicated as follows: DF: Distrito Federal; GO: Goiás; MS: Mato Grosso do Sul; MT: Mato Grosso; AC: Acre; AM: Amazonas; AP: Amapá; PA: Pará; RO: Rondônia; RR: Roraima; TO: Tocantins; AL: Alagoas; BA: Bahia; CE: Ceará; MA: Maranhão; PB: Paraíba; PE: Pernambuco; PI: Piauí; RN: Rio Grande do Norte; SE: Sergipe; PR: Paraná; RS: Rio Grande do Sul; SC: Santa Catarina; ES: Espírito Santo; MG: Minas Gerais; RJ: Rio de Janeiro; SP: São Paulo. Brazilian states in each region are as follows: South: RS, PR, and SC; North: AC, AP, AM, PA, RO, RR, and TO; Southeast: MG, SP, RJ and ES; Midwest: MT, MS, GO and DF; Northeast: AL, BA, CE, MA, PB, PE, PI, RN and SE. (TIFF 261 kb)

Additional file 2: **Table S1.** Reported cases and incidence of dengue, chikungunya, and Zika, in Brazil, in 2016. Incidence rates are indicated per 100,000 inhabitants. Data regarding the total reported cases (absolute numbers) and the incidence rates of Zika, chikungunya and dengue in different regions of Brazil were retrieved from official bulletins and tabulated. The bulletin published by Brazilian Ministry of Health, containing the data above is available at: http://combateaedes.saude.gov.br/images/boletins-epidemiologicos/2016-Dengue_Zika_Chikungunya-SE52.pdf (Accessed 26 Jan 2018). The percentages of reported cases of Zika, chikungunya and dengue were estimated per region (in relation to total cases reported in Brazil, in 2016), and are shown within brackets. The data presented here refer to the epidemiological weeks 1 to 52 of 2016. An epidemiological week (EW) is a standardized method of counting weeks to allow for the comparison of data year after year. By international convention EWs are counted from Sunday to Saturday. The first EW of the year ends, by definition, on the first Saturday of January. The epidemiological calendar of 2016, used by Brazilian Ministry of Health, is available at http://portalsinan.saude.gov.br/calendario-epidemiologico-2016. According to guides of Brazilian Ministry of Health, in areas with previous history of dengue virus, chikungunya virus or Zika virus transmission, during the epidemics periods, the confirmation of the majority of the cases should be performed by the clinical-epidemiological criteria, after the confirmation of viral circulation and epidemiological investigation of first cases in a given area. In general, blood collection and testing has been recommended for one to every 10 patients with suspected dengue fever, applying the same proportion to Zika and chikungunya (http://bvsms.saude.gov.br/bvs/publicacoes/guia_vigilancia_saude_volume_2.pdf). (DOC 39 kb)

Additional file 3: **Table S2.** Decrease in the incidence of Zika virus infection in Brazil, from 2016 to 2017. Incidence values are indicated per 100,000 inhabitants. *Data from epidemiological week 1 to 52/2016. **Data from epidemiological week 1 to 52/2017. Data regarding Zika virus infection incidence in different regions of Brazil were retrieved from official bulletins and tabulated. The ratio incidence of Zika virus infection cases 2016/ incidence of Zika virus infection 2017 was estimated and presented. The incidence rates presented here refer to epidemiological weeks 1 to 52 of each year: 2016 and 2017. The bulletins, published by Brazilian Ministry of Health, containing the incidence rates are available at http://combateaedes.saude.gov.br/images/boletins-epidemiologicos/2016-Dengue_Zika_Chikungunya-SE52.pdf (Accessed 26 Jan 2018), and at http://portalarquivos2.saude.gov.br/images/pdf/2018/janeiro/23 /Boletim-2018-001-Dengue.pdf (Accessed 26 Jan 2018). An epidemiological week (EW) is a standardized method of counting weeks to allow for the comparison of data year after year. By international convention EWs are counted from Sunday to Saturday. The first EW of the year ends, by definition, on the first Saturday of January. The epidemiological calendar of 2016 and 2017, used by Brazilian Ministry of Health, are available at http://portalsinan.saude.gov.br/calendario-epidemiologico-2016 and at http://portalsinan.saude.gov.br/calendario-epidemiologico?layout=edit&id=161, respectively. (DOC 33 kb)

Additional file 4: **Table S3.** Information of each participant were taken from patient’s file. * Geographic regions and state initials as follows: NORTH REGION: AC: Acre; AP: Amapá; AM: Amazonas; PA: Pará; RR: Roraima; RO: Rondônia; and TO: Tocantins. MIDWEST REGION: DF: Distrito Federal; GO: Goiás; MT: Mato Grosso; and MS: Mato Grosso do Sul. SOUTHEAST REGION: ES: Espírito Santo; MG: Minas Gerais; RJ: Rio de Janeiro; and SP: São Paulo. SOUTH REGION: PR: Paraná; RS: Rio Grande do Sul; and SC: Santa Catarina. NORTHEAST REGION: AL: Alagoas; BA: Bahia; CE: Ceará; MA: Maranhão; PB: Paraíba; PE: Pernambuco; PI: Piauí; RN: Rio Grande do Norte; and SE: Sergipe. (XLSX 154 kb)

Additional file 5: **Table S4.** Analysis of the difference of Zika virus detection in all Brazilian regions during 2015–2016. The difference of Zika virus detection is shown by comparing all regions each other (null hypothesis: Zika virus detection is equal among the regions). Southeast, Midwest, and North were more likely for the detection of Zika virus than Northeast and South regions. No differences were observed in Zika virus detection between Southeast, Midwest, and North regions, or between the South and Northeast regions. Statistical analysis was carried out using the Chi-squared test. (CI: Confidence Interval; OR: Odds Ratio; p: *p*-value). (DOC 33 kb)

## Abbreviations

CHIKV: Chikungunya virus
CI: Confidence interval
Ct: Cycle threshold
DENV: Dengue virus
EW: Epidemiological week
HP Institute: Hermes Pardini Institute
NS5: Nonstructural protein 5
OR: Odds ratio
RNA: Ribonucleic acid
RT-qPCR: Reverse transcription followed by real time polymerase chain reaction
ZIKV: Zika virus
