# Supplementary figures and images for: Screening for Zika virus RNA in sera of suspected cases: a retrospective cross-sectional study
Source: Virol J. 2018 Oct 11;15:155. doi: 10.1186/s12985-018-1070-z (PMC6180573; doi:10.1186/s12985-018-1070-z)

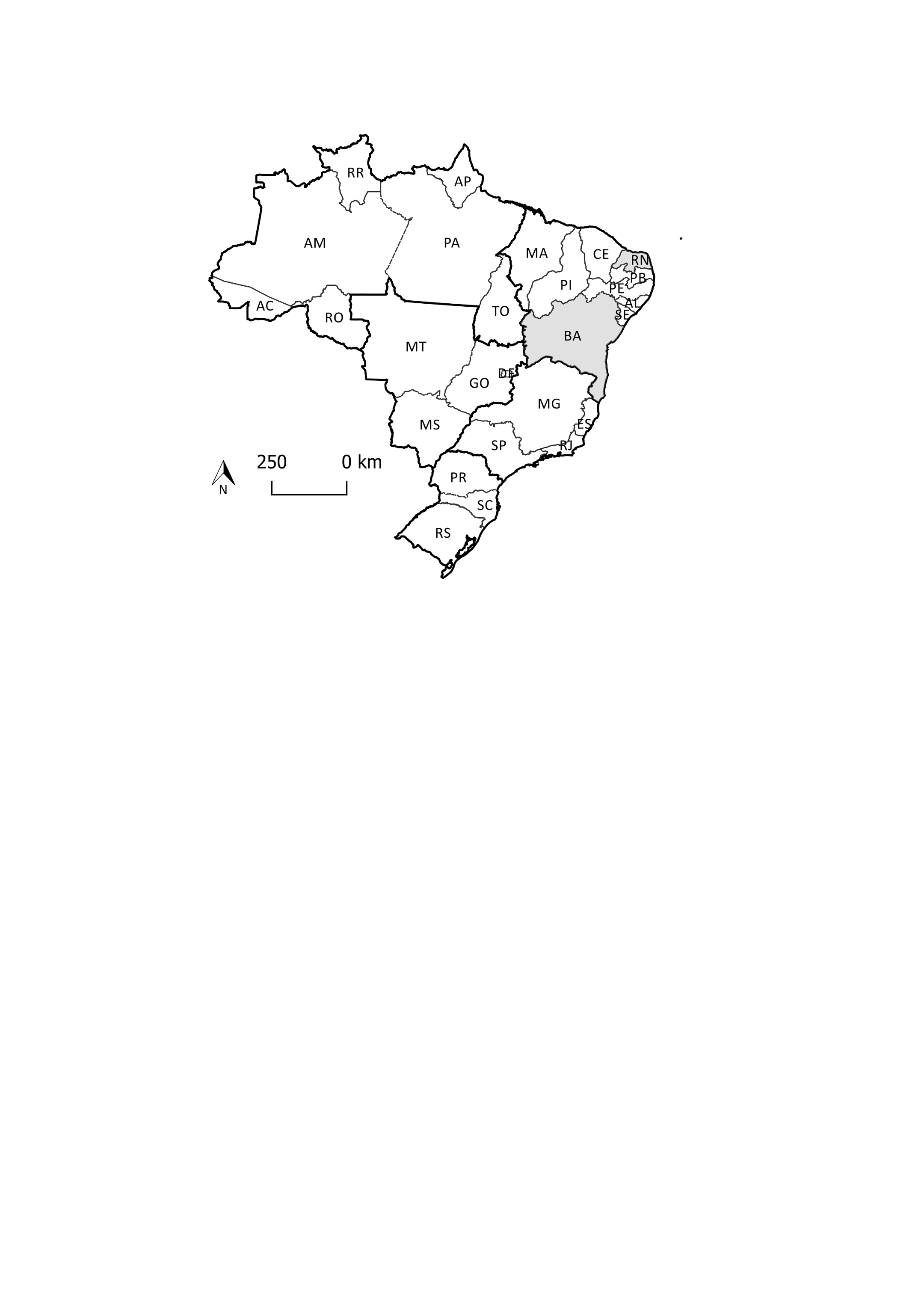

Supplement: Supplementary file 1 — Figure S1. Geopolitical map of Brazil. The Rio Grande do Norte and Bahia states, where Zika virus was first reported, is highlighted. Brazilian Federative Units are indicated as follows: DF: Distrito Federal; GO: Goiás; MS: Mato Grosso do Sul; MT: Mato Grosso; AC: Acre; AM: Amazonas; AP: Amapá; PA: Pará; RO: Rondônia; RR: Roraima; TO: Tocantins; AL: Alagoas; BA: Bahia; CE: Ceará; MA: Maranhão; PB: Paraíba; PE: Pernambuco; PI: Piauí; RN: Rio Grande do Norte; SE: Sergipe; PR: Paraná; RS: Rio Grande do Sul; SC: Santa Catarina; ES: Espírito Santo; MG: Minas Gerais; RJ: Rio de Janeiro; SP: São Paulo. Brazilian states in each region are as follows: South: RS, PR, and SC; North: AC, AP, AM, PA, RO, RR, and TO; Southeast: MG, SP, RJ and ES; Midwest: MT, MS, GO and DF; Northeast: AL, BA, CE, MA, PB, PE, PI, RN and SE. (TIFF 261 kb) [file 12985_2018_1070_MOESM1_ESM.tiff]
