# Supplementary material for: Screening for Zika virus RNA in sera of suspected cases: a retrospective cross-sectional study
Source: Virol J. 2018 Oct 11;15:155. doi: 10.1186/s12985-018-1070-z (PMC6180573; doi:10.1186/s12985-018-1070-z)
Supplement: Supplementary file 2 — Table S1. Reported cases and incidence of dengue, chikungunya, and Zika, in Brazil, in 2016. Incidence rates are indicated per 100,000 inhabitants. Data regarding the total reported cases (absolute numbers) and the incidence rates of Zika, chikungunya and dengue in different regions of Brazil were retrieved from official bulletins and tabulated. The bulletin published by Brazilian Ministry of Health, containing the data above is available at: http://combateaedes.saude.gov.br/images/boletins-epidemiologicos/2016-Dengue_Zika_Chikungunya-SE52.pdf (Accessed 26 Jan 2018). The percentages of reported cases of Zika, chikungunya and dengue were estimated per region (in relation to total cases reported in Brazil, in 2016), and are shown within brackets. The data presented here refer to the epidemiological weeks 1 to 52 of 2016. An epidemiological week (EW) is a standardized method of counting weeks to allow for the comparison of data year after year. By international convention EWs are counted from Sunday to Saturday. The first EW of the year ends, by definition, on the first Saturday of January. The epidemiological calendar of 2016, used by Brazilian Ministry of Health, is available at http://portalsinan.saude.gov.br/calendario-epidemiologico-2016. According to guides of Brazilian Ministry of Health, in areas with previous history of dengue virus, chikungunya virus or Zika virus transmission, during the epidemics periods, the confirmation of the majority of the cases should be performed by the clinical-epidemiological criteria, after the confirmation of viral circulation and epidemiological investigation of first cases in a given area. In general, blood collection and testing has been recommended for one to every 10 patients with suspected dengue fever, applying the same proportion to Zika and chikungunya (http://bvsms.saude.gov.br/bvs/publicacoes/guia_vigilancia_saude_volume_2.pdf). (DOC 39 kb) [file 12985_2018_1070_MOESM2_ESM.doc]

Table S1: Reported cases and incidence of dengue, chikungunya, and Zika, in Brazil, in 2016.

|  | Dengue 2016 | | Chikungunya 2016 | | Zika 2016 | |
| --- | --- | --- | --- | --- | --- | --- |
| Brazilian Region | Reported cases | Incidence | Reported cases | Incidence | Reported cases | Incidence |
| North | 39,011 (2.6%) | 224.7 | 8,526 (3.1%) | 48.8 | 12,973 (6.0%) | 74.2 |
| Northeast | 324,815 (21.6%) | 573.3 | 235,136 (86.5%) | 415.7 | 76,016 (35.3%) | 134.4 |
| Southeast | 858,273 (57.2%) | 1,001.2 | 24,478 (9.0%) | 28.5 | 91,053 (42.2%) | 106.2 |
| South | 72,650 (4.8%) | 250.4 | 1,884 (0.7%) | 6.4 | 993 (0.46%) | 3.4 |
| Midwest | 205,786 (13.8%) | 1,322.0 | 1,800 (0.7%) | 11.7 | 34,284 (15.9%) | 222.0 |
| Total Brazil | 1,500,535 (100.0%) | 733.4 | 271,824 (100.0%) | 133.0 | 215,319 (100.0%) | 105.3 |

Incidence rates are indicated per 100,000 inhabitants. Data regarding the total reported cases (absolute numbers) and the incidence rates of Zika, chikungunya and dengue in different regions of Brazil were retrieved from official bulletins and tabulated. The bulletin published by Brazilian Ministry of Health, containing the data above is available at: http://combateaedes.saude.gov.br/images/boletins-epidemiologicos/2016-Dengue_Zika_Chikungunya-SE52.pdf (Accessed 26 Jan 2018). The percentages of reported cases of Zika, chikungunya and dengue were estimated per region (in relation to total cases reported in Brazil, in 2016), and are shown within brackets. The data presented here refer to the epidemiological weeks 1 to 52 of 2016. An epidemiological week (EW) is a standardized method of counting weeks to allow for the comparison of data year after year. By international convention EWs are counted from Sunday to Saturday. The first EW of the year ends, by definition, on the first Saturday of January. The epidemiological calendar of 2016, used by Brazilian Ministry of Health, is available at http://portalsinan.saude.gov.br/calendario-epidemiologico-2016. According to guides of Brazilian Ministry of Health, in areas with previous history of dengue, chikungunya or Zika transmission, during the epidemics periods, the confirmation of the majority of the cases should be performed by the clinical-epidemiological criteria, after the confirmation of viral circulation of virus and epidemiological investigation of first cases in a given area. In general, blood collection and testing has been recommended for one to every 10 patients (10%) with suspected dengue fever, applying the same proportion to Zika and chikungunya *(http://bvsms.saude.gov.br/bvs/publicacoes/guia_vigilancia_saude_volume_2.pdf)*.
