# Supplementary material for: Screening for Zika virus RNA in sera of suspected cases: a retrospective cross-sectional study
Source: Virol J. 2018 Oct 11;15:155. doi: 10.1186/s12985-018-1070-z (PMC6180573; doi:10.1186/s12985-018-1070-z)
Supplement: Supplementary file 3 — Table S2. Decrease in the incidence of Zika virus infection in Brazil, from 2016 to 2017. Incidence values are indicated per 100,000 inhabitants. *Data from epidemiological week 1 to 52/2016. **Data from epidemiological week 1 to 52/2017. Data regarding Zika virus infection incidence in different regions of Brazil were retrieved from official bulletins and tabulated. The ratio incidence of Zika virus infection cases 2016/ incidence of Zika virus infection 2017 was estimated and presented. The incidence rates presented here refer to epidemiological weeks 1 to 52 of each year: 2016 and 2017. The bulletins, published by Brazilian Ministry of Health, containing the incidence rates are available at http://combateaedes.saude.gov.br/images/boletins-epidemiologicos/2016-Dengue_Zika_Chikungunya-SE52.pdf (Accessed 26 Jan 2018), and at http://portalarquivos2.saude.gov.br/images/pdf/2018/janeiro/23 /Boletim-2018-001-Dengue.pdf (Accessed 26 Jan 2018). An epidemiological week (EW) is a standardized method of counting weeks to allow for the comparison of data year after year. By international convention EWs are counted from Sunday to Saturday. The first EW of the year ends, by definition, on the first Saturday of January. The epidemiological calendar of 2016 and 2017, used by Brazilian Ministry of Health, are available at http://portalsinan.saude.gov.br/calendario-epidemiologico-2016 and at http://portalsinan.saude.gov.br/calendario-epidemiologico?layout=edit&id=161, respectively. (DOC 33 kb) [file 12985_2018_1070_MOESM3_ESM.doc]

Table S2: Decrease in the incidence of Zika virus infection in Brazil, from 2016 to 2017.

| Brazilian Region | Zika virus infection incidence | | |
| --- | --- | --- | --- |
|  | 2016* | 2017** | Ratio 2016/2017 |
| North | 74.2 | 12.4 | 5.8 |
| Northeast | 134.4 | 9.3 | 14.2 |
| Southeast | 106.2 | 4.3 | 25.0 |
| South | 3.4 | 0.3 | 10.0 |
| Midwest | 222.0 | 39.3 | 5.5 |

Incidence values are indicated per 100,000 inhabitants. *Data from epidemiological week 1 to 52/2016. **Data from epidemiological week 1 to 52/2017. Data regarding Zika virus infection incidence in different regions of Brazil were retrieved from official bulletins and tabulated. The ratio incidence of Zika virus infection cases 2016/ incidence of Zika virus infection 2017 was estimated and presented. The incidence rates presented here refer to epidemiological weeks 1 to 52 of each year: 2016 and 2017. The bulletins, published by Brazilian Ministry of Health, containing the incidence rates are available at http://combateaedes.saude.gov.br/images/boletins-epidemiologicos/2016-Dengue_Zika_Chikungunya-SE52.pdf (Accessed 26 Jan 2018), and at http://portalarquivos2.saude.gov.br/images/pdf/2018/janeiro/23 /Boletim-2018-001-Dengue.pdf (Accessed 26 Jan 2018). An epidemiological week (EW) is a standardized method of counting weeks to allow for the comparison of data year after year. They are used throughout the world and by international convention EWs are counted from Sunday to Saturday. The first EW of the year ends, by definition, on the first Saturday of January. The epidemiological calendar of 2016 and 2017, used by Brazilian Ministry of Health, are available at http://portalsinan.saude.gov.br/calendario-epidemiologico-2016 and at http://portalsinan.saude.gov.br/calendario-epidemiologico?layout=edit&id=161, respectively.
