# Supplementary material for: Screening for Zika virus RNA in sera of suspected cases: a retrospective cross-sectional study
Source: Virol J. 2018 Oct 11;15:155. doi: 10.1186/s12985-018-1070-z (PMC6180573; doi:10.1186/s12985-018-1070-z)
Supplement: Supplementary file 5 — Table S4. Analysis of the difference of Zika virus detection in all Brazilian regions during 2015–2016. The difference of Zika virus detection is shown by comparing all regions each other (null hypothesis: Zika virus detection is equal among the regions). Southeast, Midwest, and North were more likely for the detection of Zika virus than Northeast and South regions. No differences were observed in Zika virus detection between Southeast, Midwest, and North regions, or between the South and Northeast regions. Statistical analysis was carried out using the Chi-squared test. (CI: Confidence Interval; OR: Odds Ratio; p: p-value). (DOC 33 kb) [file 12985_2018_1070_MOESM5_ESM.doc]

Table S4. Analysis of the difference of *Zika virus* detection (or occurrence) in all Brazilian regions during 2015–2016

| Regions | North | Northeast | Midwest | Southeast | South |
| --- | --- | --- | --- | --- | --- |
| North |  | p <0.000001  OR: 4.59  CI95%: 2.75–7.64 | p = 1 | p = 0.4 | p = 0.00001  OR: 5.35  95% CI: 2.32–12.36 |
| Northeast | p <0.000001  OR: 4.59  95% CI: 2.75–7.64 |  | p <0.000001  OR: 5.8  95% CI:  3.81–8.90 | p <0.000001  OR: 3.9  95% CI:  2.63–5.78 | p = 0.83 |
| Midwest | p = 1 | p <0.000001  OR: 5.8  95% CI: 3.81 - 8.90 |  | p = 0.25 | p <000001  OR: 5.39  95% CI: 2.45 - 11.83 |
| Southeast | p = 0.4 | p <0.000001  OR: 3.9  95% CI: 2.63–5.78 | p = 0.25 |  | p = 0.000003  OR: 4.5  95% CI: 2.10–9.84 |
| South | p = 0.00001  OR: 5.35  95% CI: 2.32–12.36 | p = 0.83 | p <000001  OR: 5.39  95% CI:  2.45–11.83 | p = 0.000003  OR: 4.5  95% CI:  2.10–9.84 |  |

The difference of *Zika virus* detection is shown by comparing all regions each other (null hypothesis: *Zika virus* detection is equal among the regions). Southeast, Midwest, and North were more likely for the occurrence of *Zika virus* than Northeast and South regions. No differences were observed in *Zika virus* occurrence between Southeast, Midwest, and North regions, or between the South and Northeast regions. Statistical analysis was carried out using the Chi-squared test. (CI: Confidence Interval; OR: Odds Ratio; p: p-value).
